# Supplementary material for: Efficient recognition of facial expressions does not require motor simulation
Source: eLife. 2020 May 4;9:e54687. doi: 10.7554/eLife.54687 (PMC7217693; doi:10.7554/eLife.54687)
Supplement: Supplementary file 1. [file elife-54687-supp1.docx]

|  | **Sex** | **Age (years)** | **Education (years)** | **Neurological history** | **Psychiatric history** | **Surgery linked to Moebius** | **Therapy linked to Moebius** |
| --- | --- | --- | --- | --- | --- | --- | --- |
| **IMS1** | F | 37 | 4 | Peripheral neuropathy: hyper sensibility to touch and pressure; tinnitus | Past history of depression | Right foot orthopedic surgery (clubfoot correction) | Speech therapy during childhood (1 year), around 24 y (1 year), currently (6 months prior) |
| **IMS2** | F | 36 | 3 | None reported | None reported | Correction of strabismus and right-hand syndactyly | None reported |
| **IMS3** | M | 19 | 2 | None reported | None reported | Feet orthopedic surgery (clubfeet correction) | Speech therapy (4 to 15 y) |
| **IMS4** | F | 43 | 1 | Mild head concussion (18 y) | None reported | More than 10 facial surgeries from 16 to 21 y; Ophthalmic surgery to improve eye lid closure; smile restoration surgery^1, 2^ | Speech therapy (6 to 7 y) |
| **IMS5** | F | 31 | 2 | None reported | None reported | Lachrymal duct surgery and ophthalmic surgery to improve eye lid closure | Speech therapy (2 to 14 y) |
| **IMS6** | F | 19 | 0 | 3 days of artificial coma at 12 y (viral infection) | Past history of depression | Smile restoration surgery^1, 2^ (13 y) | Speech therapy, (6 – ongoing) |
| **IMS7** | M | 31 | 2 | Hypopituitarism | Past history of depression | Several smile restoration surgeries; Correction of strabismus | None reported |
| **IMS8** | F | 21 | 3 | None reported | None reported | None reported | None reported |
| **IMS9** | F | 15 | 0 | None reported | None reported | Smile restoration surgery on the left side at 8y; Correction of strabismus | None reported |
| **IMS10** | F | 20 | 0 | None reported | None reported | None reported | Speech therapy (6 to 16 y) |
| **IMS11** | M | 33 | 3 | Two epileptic seizures 2-3 years prior (unknown origin). Lamictal 100 mg/day | Past history of depression | Feet orthopedic surgery (clubfeet correction) | Speech therapy (4 to 10 y). Physical therapy (lower limbs). |
