# Supplementary material for: Efficient recognition of facial expressions does not require motor simulation
Source: eLife. 2020 May 4;9:e54687. doi: 10.7554/eLife.54687 (PMC7217693; doi:10.7554/eLife.54687)
Supplement: Supplementary file 2. [file elife-54687-supp2.docx]

|  | Facial Action Units^1^ | | | | | | | | | | | | |
| --- | --- | --- | --- | --- | --- | --- | --- | --- | --- | --- | --- | --- | --- |
|  | AU01 | AU02 | AU04 | AU05 | AU06 | AU07 | AU09 | AU12 | AU15 | AU16 | AU20 | AU23 | AU26 |
| Happiness | /^2^ | / | / | / | none^3^ | / | / | 8^4^ | / | / | / | / | / |
| Sadness | none | / | none | / | / | / | / | / | 10 | / | / | / | / |
| Surprise | none | none | / | 8, 10 | / | / | / | / | / | / | / | / | 8, 10 |
| Fear | none | none | none | 8, 10 | / | none | / | / | / | / | none | / | 8, 10 |
| Anger | / | / | none | 8, 10 | / | none | / | / | / | / | / | none | / |
| Disgust | / | / | / | / | / | / | none | / | 10 | none | / | / | / |

^1^ AU01: Inner Brow Raiser; AU02: Outer Brow Raiser; AU04: Brow Lowerer; AU05: Upper lid raiser; AU06: Cheek Raiser; AU07: Lid Tightener; AU09: Nose wrinkle; AU12: Lip Corner Puller; AU15: Lip Corner Depressor; AU16: Lower Lip Depressor; AU20: Lip Stretcher; AU23: Lip Tightener; AU26: Jaw Drop (Ekman, Friesen & Hager, 2002). ^2^ AU not involved in the typical expression of this emotion. ^3^ AU absent from the repertoire of the three IMS. ^4^ AU present in the repertoire of this/these IMS.
