# Supplementary material for: Efficient recognition of facial expressions does not require motor simulation
Source: eLife. 2020 May 4;9:e54687. doi: 10.7554/eLife.54687 (PMC7217693; doi:10.7554/eLife.54687)
Supplement: Transparent reporting form [file elife-54687-transrepform.docx]

***eLife’s* transparent reporting form**

We encourage authors to provide detailed information *within their submission* to facilitate the interpretation and replication of experiments. Authors can upload supporting documentation to indicate the use of appropriate reporting guidelines for health-related research (see [EQUATOR Network](http://www.equator-network.org/%20)), life science research (see the [BioSharing Information Resource](https://biosharing.org/)), or the [ARRIVE guidelines](http://www.plosbiology.org/article/info:doi/10.1371/journal.pbio.1000412) for reporting work involving animal research. Where applicable, authors should refer to any relevant reporting standards documents in this form.

If you have any questions, please consult our Journal Policies and/or contact us: [editorial@elifesciences.org](mailto:editorial@elifesciences.org).

**Sample-size estimation**

- You should state whether an appropriate sample size was computed when the study was being designed
- You should state the statistical method of sample size computation and any required assumptions
- If no explicit power analysis was used, you should describe how you decided what sample (replicate) size (number) to use

Please outline where this information can be found within the submission (e.g., sections or figure legends), or explain why this information doesn’t apply to your submission:

In this study, we investigated whether it is possible for individuals born with congenital facial paralysis to achieve typically efficient facial expression recognition. Given the heterogeneity of symptoms frequently associated with congenital facial paralysis, this research relies on a single-case design in neuropsychology, which aims at using patterns of associations and dissociations in an individual with atypical sensorimotor development (or brain damage) to inform our understanding of normal cognitive functioning. We conducted 11 single-case studies and compared each case’s performance to that of 25 control participants.

This was determined on the following bases: (1) the maximal number of rare individuals with congenital facial paralysis that we would be able to recruit and test in 3 years and (2) 25 control participants. (Page 7 lines 7-22). Inferences from single cases were allowed by the use of Crawford and Howell’s (1998) modified t-test. With a sample size of 25 control participants, this method has a good power to detect a deficit (Crawford & Garthwaite, 2006). To increase the power to detect a possible deficit, and severely decrease the risk of false negatives, we set the threshold for a possible deficit at *p* < 0.2. (as explained Page 8 lines 3-15).

References:

Crawford, J.R., & Howell, D.C. (1998). Comparing an individual’s test score against norms derived from small samples. *Clin Neuropsychol, 12*(4), 482-486.

Crawford, J.R., & Garthwaite, P.H. (2006). Methods of testing for a deficit in single-case studies: Evaluation of statistical power by Monte Carlo simulation. *Cognitive Neuropsychology*, *23*(6), 877-904.

**Replicates**

- You should report how often each experiment was performed
- You should include a definition of biological versus technical replication
- The data obtained should be provided and sufficient information should be provided to indicate the number of independent biological and/or technical replicates
- If you encountered any outliers, you should describe how these were handled
- Criteria for exclusion/inclusion of data should be clearly stated
- High-throughput sequence data should be uploaded before submission, with a private link for reviewers provided (these are available from both GEO and ArrayExpress)

Please outline where this information can be found within the submission (e.g., sections or figure legends), or explain why this information doesn’t apply to your submission:

All the experiments were performed once by each subject. All the experiments that we performed are reported in the paper (See Figure 1). All the data that we collected are reported in the paper (see Figure 1). No data was excluded.

The performances of one control participant in Experiment 1 and of another control participant in Experiment 2 were considered “outliers” because they were below 2 standard deviations from the control participants’ average in these experiments. The rationale for treating these data as outliers is described on page 8 (lines 3-15). These performances are clearly indicated by an asterisk (*) on Figure 1.

**Statistical reporting**

- Statistical analysis methods should be described and justified
- Raw data should be presented in figures whenever informative to do so (typically when N per group is less than 10)
- For each experiment, you should identify the statistical tests used, exact values of N, definitions of center, methods of multiple test correction, and dispersion and precision measures (e.g., mean, median, SD, SEM, confidence intervals; and, for the major substantive results, a measure of effect size (e.g., Pearson's r, Cohen's d)
- Report exact p-values wherever possible alongside the summary statistics and 95% confidence intervals. These should be reported for all key questions and not only when the p-value is less than 0.05.

Please outline where this information can be found within the submission (e.g., sections or figure legends), or explain why this information doesn’t apply to your submission:

- Statistical analysis methods are described and justified on page 8 (lines 3-15), 14 (lines 17-19) and 15-16 (lines 10-17 and 1-7).

- Raw data are presented in Figure 1.

- The statistical tests used and the values of N are clearly identified in the result section (pages 13-17). The average performance of the control participants, the distribution of their performance and the performance of the individuals with congenital facial paralysis are all clearly indicated on Figure 1. Measure of effect size is reported whenever relevant (P 16 lines 9-22).

- In this paper, we discuss the results of statistical analyses comparing 11 individuals with congenital paralysis and a control group in 8 experiments. Given the number of tests, we decided no to report the exact p-value of each test. Instead, we opted to display the raw data for all the participants in all the experiments (Figure 1) together with symbols indicating participants meeting statistical criteria relevant for the study: a small circle (°) indicates participants with congenital paralysis with a “normotypical” score (at least above 0.85 standard deviation below the controls’ mean performance) and an asterisk (*) indicates a participants with an abnormally low score (below 2 SD from the other control participants) (see the legend of Figure 1).

(For large datasets, or papers with a very large number of statistical tests, you may upload a single table file with tests, Ns, etc., with reference to sections in the manuscript.)

**Group allocation**

- Indicate how samples were allocated into experimental groups (in the case of clinical studies, please specify allocation to treatment method); if randomization was used, please also state if restricted randomization was applied
- Indicate if masking was used during group allocation, data collection and/or data analysis

Please outline where this information can be found within the submission (e.g., sections or figure legends), or explain why this information doesn’t apply to your submission:

Not applicable.

**Additional data files (“source data”)**

- We encourage you to upload relevant additional data files, such as numerical data that are represented as a graph in a figure, or as a summary table
- Where provided, these should be in the most useful format, and they can be uploaded as “Source data” files linked to a main figure or table
- Include model definition files including the full list of parameters used
- Include code used for data analysis (e.g., R, MatLab)
- Avoid stating that data files are “available upon request”

Please indicate the figures or tables for which source data files have been provided:

Data and stimulus materials are publicly available and can be accessed on the Open

Science Framework platform (https://osf.io/8t4fv/?view_only=85c15cafe5d94bb6a5cff2f09a6ef56d)
